# Nest signature changes throughout colony cycle and after social parasite invasion in social wasps

**DOI:** 10.1371/journal.pone.0190018

**Published:** 2017-12-19

**Authors:** Marta Elia, Giuliano Blancato, Laura Picchi, Christophe Lucas, Anne-Geneviève Bagnères, Maria Cristina Lorenzi

**Affiliations:** 1 Department of Life Sciences and Systems Biology, University of Turin, Torino, Italy; 2 I.R.B.I. – UMR 7261 CNRS – Université de Tours, Faculté des Sciences, Parc Grandmont, Tours, France; 3 CEFE UMR 5175, CNRS – Université de Montpellier – Université Paul Valéry Montpellier – EPHE, Montpellier, France; 4 LEEC-Laboratoire d'Ethologie Expérimentale et Comparée, Université Paris 13, Sorbonne Paris Cité, Villetaneuse, France; Universidade de Sao Paulo Faculdade de Filosofia Ciencias e Letras de Ribeirao Preto, BRAZIL

## Abstract

Social insects recognize their nestmates by means of a cuticular hydrocarbon signature shared by colony members, but how nest signature changes across time has been rarely tested in longitudinal studies and in the field. In social wasps, the chemical signature is also deposited on the nest surface, where it is used by newly emerged wasps as a reference to learn their colony odor. Here, we investigate the temporal variations of the chemical signature that wasps have deposited on their nests. We followed the fate of the colonies of the social paper wasp *Polistes biglumis* in their natural environment from colony foundation to decline. Because some colonies were invaded by the social parasite *Polistes atrimandibularis*, we also tested the effects of social parasites on the nest signature. We observed that, as the season progresses, the nest signature changed; the overall abundance of hydrocarbons as well as the proportion of longer-chain and branched hydrocarbons increased. Where present, social parasites altered the host-nest signature qualitatively (adding parasite-specific alkenes) and quantitatively (by interfering with the increase in overall hydrocarbon abundance). Our results show that 1) colony odor is highly dynamic both in colonies controlled by legitimate foundresses and in those controlled by social parasites; 2) emerged offspring contribute little to colony signature, if at all, in comparison to foundresses; and 3) social parasites, that later mimic host signature, initially mark host nests with species-specific hydrocarbons. This study implies that important updating of the neural template used in nestmate recognition should occur in social insects.

## Introduction

Social insect colonies are closed societies in which intruders are rejected, and thus, the integrity and the resources of the colonies are maintained [1]. Social insects are able to discriminate between nestmates and non-nestmate [2, 3], and the cuticular hydrocarbons are the main cues used for the discrimination [4].

Cuticular hydrocarbons cover the body of individuals and compose the chemical signature [2]. Typically, qualitative differences in the chemical signatures discriminate between species, while quantitative differences are found among colonies of the same species in ants, bees and wasps [5, 6, 7, 8, 9]. Cuticular hydrocarbons produced by the insects are also found on their nests, including the wall of the underground nests of ants, e.g. [10], the waxy comb of bees [11] or the paper nests of social wasps [12].

The paper nest, and the hydrocarbons that cover its surface, are especially relevant in the social biology of *Polistes* social wasps. During nest foundation, wasp foundresses mark their nests by stroking the abdomen on the nest surface [5, 13]. Possibly as a result of this marking behaviour, which might imply foundresses depositing their signature on the nest, foundresses and nests share the same hydrocarbon blends [5, 12, 14]. Wasps recognize their nests using nest odors [15, 16].

Further support for the crucial role of the nest—and the chemicals on its surface—in wasp social biology comes from the observations on the behaviour of the social parasites of paper wasps. When *inter*- and *intra*specific social parasites invade their colonies, parasites spend time rubbing their abdomens on the host nest surface [17, 18]. The host nest-marking by *intra*specific social parasites has been associated with changes in host nest odors; as parasites establish in host colonies, the chemical signature of the host nests becomes biased towards that of the *intra*specific parasites themselves [5, 19, 20]. Similarly, obligate social parasite *Polistes sulcifer* contaminated artificial host nests made of plastic and aluminium with parasite-specific compounds [21]. These marking activities by both foundresses and social parasites are reasonably correlated with the role that the paper nest has in relation to the learning of colony odor. Indeed, there is a large amount of experimental evidence that proves that social wasps use the nest—and the chemicals therein—as a reference for their colony odor [22].

The exposure of newly emerged wasps to the colony odor deposited on the nest material is a fundamental step in the development of the recognition abilities and in the formation of the “template” for the nestmate recognition, that is, the neural representation of the colony odor stored in the memory [6]. Social wasps tolerate individuals with a chemical signature similar to the template formed, and attack those that are different [22, 23]. Young wasps exposed to their natal nests soon after the eclosion developed sufficiently good nestmate discrimination abilities, and the learning process was completed four hours later (reviewed in [22]), but was functional only if the nest was covered by hydrocarbons [24] (although the timing and other details of the learning process has been recently debated [25, 26]). In the lab, where the homogenous environment fades variations and colonies end up more similar than they were initially [27], the template formed at the emergence is still effective after months [28]. Studies on the learning of recognition cues often assume that colony odor is stable and it does not change over time, so that the learning mechanisms involved in the formation of the neural template have also been referred to as imprinting-like learning, e.g. [22].

However, colony odor might change over time as a function of variations in the physiological state of the foundress(es), and in the social structure and demography of the colony, or because diet, meteorological conditions, and natural enemies vary throughout colony cycle (e.g., [29, 30, 31]). In turn, the variation in colony odor may reflect in the signature deposited on the nest material and affect nestmate recognition mechanisms and the way the neural template is maintained and/or updated. Indeed, in the ant *Camponotus aethiops*, swapping soil between colonies caused the ants exposed to the soil from another colony to become chemically more similar (as for their cuticular hydrocarbon profile) to the foreign colony, and to receive less aggression from ants from the colony supplying the soil [10]. In the honeybee *Apis mellifera*, swapping wax-combs between colonies, demonstrate that bees exposed to non-nestmate colony odor became more tolerant towards non-nestmates i.e., they modified their template [11].

In the present research, we aimed to study whether and how the nest signature changed throughout the colony cycle as residents lived undisturbed in the wild, foraged freely and their colonies were exposed to natural enemies. Moreover, we investigated whether the changes in the social life of the colonies (e.g., offspring emergence, social parasite invasion) played a role in the variation of the nest signature. We also aimed to understand whether the foundresses were mainly responsible for nest signature, and whether the offspring contributed as well. Furthermore, the invasion of host colonies by obligate social parasites offered the opportunity to test—via a natural experiment—whether parasites interfered with the nest signature and its variations. In order to do this, we tracked the nest signature of the social paper wasp *P*. *biglumis* in the wild from nest foundation until the end of the nesting season, when residents abandoned the nests. We extended our analyses to both colonies controlled by their legitimate foundresses (free-living colonies) and to those invaded by the social parasite *P*. *atrimandibularis* (parasitized colonies).

## Methods and materials

### Model species

*Polistes biglumis* is a social paper wasp with a boreo-montane distribution [32] that in southern Europe lives in high mountain habitats. Due to the harsh environmental conditions, the colonies of *P*. *biglumis* have a short colony cycle (3–4 months) [33], which makes it easy to follow the variations of the colony signature from the foundation until the colony declines. The colony cycle begins at the end of May-beginning of June, when overwintered *P*. *biglumis* foundresses solitarily found their colonies in open mountain meadows above 1000 m a.s.l. [33]. Foundresses build their paper nests by chewing plant fibers from weathered wood, mixing it with saliva, and shaping this material in a comb, which usually consists of less than 100 cells at the peak of colony growth [33]. Foundresses use nest cells to rear their offspring, but in this species, the peripheral ring of cells is always left empty, possibly as a way to protect brood from harsh weather conditions [33, 34]. Each foundress is the only adult on her colony for about 40 days (founding phase) and the only individual responsible for nest construction and brood care, before her brood emerge starting in mid-July; adults continue to emerge throughout the summer, until the end of August/mid-September. Colonies are small, and the brood is composed of few workers, if any, and few reproductive males and females [33, 35]. Due to the short nesting season (100 days approximately), an average of less than 20 wasps per colony emerge during the whole summer [33]. Newly emerged wasps often desert the colony soon after eclosion, so that colonies are rarely inhabited by more than 10 individuals [36].

*P*. *biglumis* colonies seldom survive intact until the end of the summer, because predators, *intra*specific and *inter*specific social parasites destroy or invade them [34]. Specifically, at the end of the founding phase and before brood emergence, some colonies are invaded by the rare, obligate social parasite *P*. *atrimandibularis*. Social parasites enter the colony peacefully, enslave the host foundress and exploit the hosts for rearing their own brood [18]. Soon after host-nest invasion, social parasites eat the youngest host brood while sparing the oldest ones (more likely to become workers) [34, 37]. Parasites start to lay eggs, and then the host foundresses care for the parasite brood, helped by the host descendants, later in the season [38].

The chemical signature of the host species *P*. *biglumis* is composed of 72 saturated hydrocarbons: *n*-alkanes, laterally and centrally branched monomethylalkanes, dimethylalkanes, and trimethylalkanes, ranging from 23 to 35 carbon atoms [39, 40, 41].

The chemical signature of *P*. *atrimandibularis* social parasites includes up to 46 saturated and unsaturated hydrocarbons with chain length up to 28 carbon atoms before host nest invasion, and extends up to 35 carbon-atoms one month after host nest invasion, when parasites chemically mimic their hosts [42]. After that, hosts recognize the resident parasites as nestmates [43]. The *pre*-invasion chemical signature of the social parasites is rich in several unsaturated compounds, such as (*Z)*-9-pentacosene, (*Z*)-9-nonacosene and (*Z*)-9-heptacosene (the latter being the most abundant), and nonacosene being the only compound with 29 carbon atoms in the *pre*-invasion chemical signature, which disappears in the *post*-invasion chemical signature during mimicry [40, 42].

### Ethical standards

The collection of colonies and the experiments performed comply with the current laws in Italy and France. No specific permits were required for the collection, nor for collection location. The species used in the experiments are not endangered or protected in Italy and France.

### Sample collection

In order to describe the chemical variation of *P*. *biglumis* colony signature over time and identify whether parasitized nests differ in this respect from free-living ones, we followed the fate of 129 *P*. *biglumis* colonies in the wild from their foundation at the beginning of June until their decline at the end of August.

The study was carried out in two populations in the Western Alps (Montgenèvre: Hautes Alpes, France, 44°55’N, 6°43’E; Ferrere: Valle Stura di Demonte, Cuneo, Italy 44°22’N, 6°57’E) in the summer 2014 (5 June– 25 August 2014).

The first time we checked a nest, the foundress was marked on the thorax with enamel paint for individual identification (n = 129). Then, we visited colonies weekly until their decline. At each census, we checked whether the foundress was present and whether a *P*. *atrimandibularis* parasite female was also present, which would indicate that the colony was parasitized. If present, we marked the parasite for individual identification. Invasions by *P*. *atrimandibularis* parasites occurred in 40 colonies out of 129 between 10 June and 10 July. At each census, we also counted the number of brood (eggs, larvae and pupae) and we noted whether the offspring had emerged in order to identify the colony phase (before or after brood emergence). Finally, at each visit, we cut a fragment of nest paper from the external lateral wall of each nest (approx. 1x1 cm^2^) using scissors (scissors were washed with solvent before each use). Each week, we took the fragment from a different side of the nest, to reduce disturbance on the nests. For the same reason, we did not collect nest fragments when nests were smaller than 15 cells. To test whether nest signature was homogenous on the nest surface, we collected two fragments simultaneously from nine nests chosen at random from the 129 nests: one fragment from the right side and one from the left side.

At sampling, the nest fragments were kept separately in glass vials, brought to the laboratory and frozen at -20°C.

Sample size varied during the summer because some nests were discovered later during the season, and others were killed by enemies (e.g., birds, small mammals but also humans and cows) at different time during colony cycle. Finally, some nests were missing in some surveys for logistical reasons. In total, excluding 74 nests invaded by *intra*specific social parasites, or where we were uncertain whether the female was the foundress or an *intra*specific parasite, we included in our analyses 15 free-living nests (i.e., nests controlled by the foundress for the entire colony cycle) and 40 parasitized nests (i.e., nests invaded by the obligate social parasites). From these 55 nests, we collected 193 samples (66 fragments from free-living nests, 1–8 time points per nest; 127 fragments from parasitized nests, 1–9 time points per nest).

Nest fragments were weighed with a precision balance (Precisa 125A) (fragment weight: 3.01 ± 0.05 mg) to correct the concentrations of hydrocarbons by the weight of fragments.

### Chemical analyses

We extracted hydrocarbons by dipping each nest fragment separately in 500 μl of heptane for 60 sec twice, thus obtaining 1000 μl of solution for each nest fragment. Extracts were dried under a gentle nitrogen stream and re-dissolved in 100 μl of heptane containing *n*-C20 as an internal standard.

We injected 2 μl of each extract (which included 800 ng of *n*-C20) into a gas chromatograph with the flame ionization detection (GC-FID: Agilent Technologies 6850 equipped with a J&W HP-1 nonpolar-capillary-column, 30 m L x 0.32 mm ID x 0.25 μm PT) for the quantification of the hydrocarbons.

We injected another 2 μl of the extracts into a gas chromatograph-mass spectrometer (Agilent Technologies 7000C GC-MS Triple Quad equipped with a Zebron ZB-5HT capillary-column, 30 m L x 0.25 ID x 0.25 μm PT) for the identification of the hydrocarbons.

We used the same oven temperature program for both GC-FID and GC-MS (from 70°C to 150°C at 30°C/min and from 150°C to 320°C at 5°C/min; 10 min at the 320°C final temperature; carrier gas was helium at 1 bar and 50 ml/min flux; injector temperature was 250°C). Results were registered with the Agilent ChemStation program for GC-FID and Agilent Mass Hunter Workstation program for GC-MS.

### Identification and quantification of compounds

Most of the compounds have been previously described in Bagnères et al. [42] and Lorenzi et al. [39] using GC-MS in EI and CI modes; determination was then completed in Uboni et al. [40] and Bonelli et al. [41]. Verification of their presence was performed comparing the diagnostic ions and the M-15 of each peak by the GC-MS apparatus described above. For more details on structure and analysis of insect hydrocarbons see [44]. For quantification, peak areas were integrated using the GC ChemStation software and corrected manually.

### Statistical analyses

Following Bagnères et al. [45] and Lorenzi et al. [31], we quantified the total concentration of hydrocarbons on the nest fragments as the sum of all peak areas relative to the area of the internal standard (*n*-C20), which corresponded to 800 ng, and we divided this value by nest fragment weight (ng of hydrocarbons/mg of nest-fragment).

Then, we tested whether the nest hydrocarbon concentrations on the nest varied over time in free-living and parasitized nests using Generalized Linear Mixed Models (GLMM) (normal distribution, log link). Time was used as a fixed effect (each time period included 14 days and two nest samplings). The other fixed effects were nest status, population (Montgenèvre and Ferrere), brood (as a measure of the nest size), and colony phase (before or after brood emergence). Nest identity was used as a random factor; the random slope for time was included in the model as a random effect (nest identity as a subject; time as an effect).

In order to investigate the variation in the nest signature, we transformed the relative proportions of hydrocarbons using the log-ratio-transformation (natural log of the proportion of each peak divided by the geometric mean of the proportions of alkanes) according to Aitchison [46]. We reduced the number of variables (log-ratio-transformed hydrocarbon proportions) by performing a Principal Component Analysis (PCA) on all the 67 hydrocarbons extracted from the nest fragments (PCA was based on correlation matrix, Varimax rotation). The PCA produced 14 Principal Components (PCs) (eigenvalues > 1; cumulative variance explained 80.94%). Then, we tested whether there were variations over time in the first three PCs (which were the PCs that cumulatively explained at least 51% of the variance) and by nest status using GLMMs (normal distribution, identity link). The other fixed effects were population, brood, and colony-cycle phase. A random factor with a random slope was included in the model (nest identity as a subject; time as an effect).

Finally, we tested whether the proportion of longer-chain hydrocarbons and that of branched hydrocarbons varied with time and nest status. We classified hydrocarbons as shorter-chain hydrocarbons if they had a chain length from C22 to C28 carbon atoms (chain length < 29 carbon atoms: peak 1–25; these hydrocarbons are present in the chemical signature of hosts and *pre*-invasion parasites). We classified hydrocarbons as longer-chain hydrocarbons if they had a chain length ≥ 29 carbon atoms (peak 26–63; these hydrocarbons are present in the chemical signature of hosts and *post*-invasion parasites). We built two different GLMMs to test the variation in the (arcsine-square-root transformed) proportion of longer-chain hydrocarbons and that of branched hydrocarbons (normal distribution and identity link), which were entered as the response variables. Time, nest status, population, brood and colony-cycle phase were the fixed effects in both models; nest identity was included as a random factor. In the GLMM on the proportion of longer-chain hydrocarbons we also included time as a random effect (this was not done in the other model because the model failed to converge).

The preliminary GLMMs included all biologically reasonable interactions and terms. Then, we dropped the least non-significant interactions and terms and calculated the reduced models.

Because most colonies were killed by predators during the summer, we could only sample few colonies in August (n = 12 fragments). In order to test whether the variation in the chemical traits was driven by the values in late August, we also ran all Models using a reduced dataset that excluded late August data. These analyses yielded approximately similar statistical results as those of the complete data set, which we report in the Results section.

Preliminary analyses showed that nest side did not explain the variation in the traits describing nest signature, and, therefore, this factor was not included in the models presented in the paper. This result suggests that the signature was spatially homogeneous on the nest surface, as supported by the consistent results that we obtained by sampling nests once on the right side, and once on the left side.

Descriptive statistics are mean ± S.E. Statistical analyses were performed in IBM SPSS Statistic 22.0.

## Results

The nest signature was composed of 67 peaks and was a mixture of linear alkanes, monomethylalkanes, dimethylalkanes, trimethylalkanes and alkenes, ranging from 23 to 35 carbon-chain length. The hydrocarbons found on the nest paper of free-living colonies were the same as those previously reported for *P*. *biglumis* wasps [39, 40, 41], whereas additional hydrocarbons were found on the nest paper of parasitized colonies at certain sampling times during the season. These additional compounds were unsaturated, parasite-specific, and previously identified [40, 42] (see below) (Table 1).

**Table 1:** List of the hydrocarbons on free-living and parasitized nests.

PEAKS	HYDROCARBONS	FREE-LIVING NESTS	PARASITIZED NESTS (SEE TEXT)	PEAKS	HYDROCARBONS	FREE-LIVING NESTS	PARASITIZED NESTS (SEE TEXT)
1	*n*-Tricosane	✓	✓	30	11,15-+9,15-+9,17-+9,19-Dimethylnonacosane	✓	✓
2	*n*-Tetracosane	✓	✓	31	3-Methylnonacosane	✓	✓
3	*n*-Pentacosane	✓	✓	32	5,11-+5,13-Dimethylnonacosane	✓	✓
3a	9-Pentacosene		✓	33	5,9-Dimethylnonacosane	✓	✓
4	11-+13-Methylpentacosane	✓	✓	34	*n*-Triacontane	✓	✓
5	7-Methylpentacosane	✓	✓	35	3,9-+3,11-+3,13-Dimethylnonacosane	✓	✓
6	5-Methylpentacosane	✓	✓	36	9-+10-+11-+12-+13-+14-Methyltriacontane	✓	✓
7	11,15-Dimethylpentacosane	✓	✓	37	2-Methyltriacontane	✓	✓
8	3-Methylpentacosane	✓	✓	38	9,19-Dimethyltriacontane	✓	✓
9	*n*-Hexacosane	✓	✓	39	4,12-Dimethyltriacontane	✓	✓
9a	3,11-+3,13-+3,15-Dimethylpentacosane	✓	✓	40	*n*-Hentriacontane	✓	✓
10	12-Methylhexacosane	✓	✓	41	9-+11-+13-+15-Methylhentriacontane	✓	✓
11	2-Methylhexacosane	✓	✓	42	7-Methylhentriacontane	✓	✓
11a	3-Methylhexacosane	✓	✓	43	11,15-Dimethylhentriacontane	✓	✓
11b	9-Heptacosene		✓	44	9,17-+9,19-+9,21-Dimethylhentriacontane	✓	✓
12	*n*-Heptacosane	✓	✓	45	5,11-+5,13-Dimethylhentriacontane	✓	✓
13	9-+11-+13-Methylheptacosane	✓	✓	46	*n*-Docotriacontane	✓	✓
14	7-Methylheptacosane	✓	✓	47	3,9-+3,11-+3,13-Dimethylhentriacontane	✓	✓
15	5-Methylheptacosane	✓	✓	48	10-+11-+12-+13-+14-Methydocotriacontane	✓	✓
16	11,15-Dimethylheptacosane	✓	✓	49	11,15-+11,19-+11,21-Dimethyldocotriacontane	✓	✓
17	3-Methylheptacosane	✓	✓	50	2-Methyldocotriacontane	✓	✓
18	5,11-+5,13-Dimethylheptacosane	✓	✓	52	*n*-Tritriacontane	✓	✓
19	*n*-Octacosane	✓	✓	53	9-+11-+13-+15-+17-Methyltritriacontane	✓	✓
20	3,9-+3,11-+3,13-Dimethylheptacosane	✓	✓	54	13,21-+11,15-Dimethytritriacontane	✓	✓
21	8-+11-+12-+13-+14-Methyloctacosane	✓	✓	55	9,19-+9,21-+9,23-Dimethyltritriacontane	✓	✓
22	4-Methyloctacosane	✓	✓	56	11,17,21-+11,17,23-Trimethyltritriacontane	✓	✓
23	2-Methyloctacosane	✓	✓	57	9,13,17-+9,15,19-+9,15,21-+9,15,23-Trimethyltritriacontane	✓	✓
24	3-Methyloctacosane	✓	✓	58	*n*-Tetratriacontane	✓	✓
25	Unknown	✓	✓	59	3,9-+3,11-+3,13-+3,15-Dimethyltritriacontane	✓	✓
26a	9-Nonacosene		✓	60	10-+11-+12-+13-+15-Methyltetratriacontane	✓	✓
26	*n*-Nonacosane	✓	✓	61	2-Methyltetratriacontane	✓	✓
27	9-+11-+13-+15-Methylnonacosane	✓	✓	62	11-+13-+15-+17-Methylpentatriacontane	✓	✓
28	7-Methylnonacosane	✓	✓	63	9,23-+11,15+11,17-Dimethylpentatriacontane	✓	✓
29	5-Methylnonacosane	✓	✓				

### Analyses of the concentration of hydrocarbons on the nests

**Fig 1:**
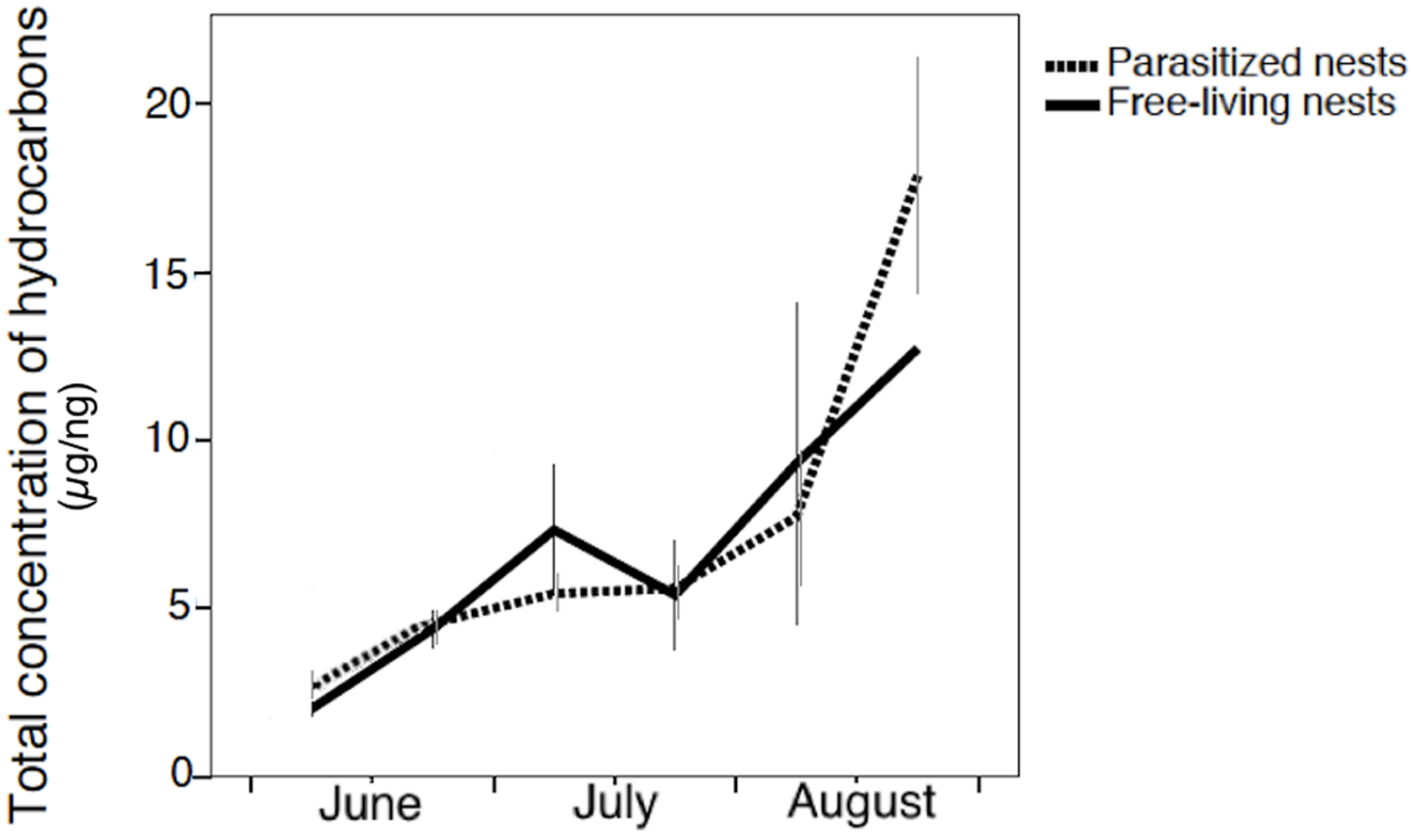
The variation in the concentration of hydrocarbons on the nest paper throughout the season in free-living nests controlled by the *P*. *biglumis* foundresses and in parasitized nests controlled by the social parasite *P*. *atrimandibularis* (in μg/mg of nest paper).

Nests had a relatively high concentration of hydrocarbons on their surface, with an average of 4.85 ± 2.57 μg of hydrocarbons per mg of nest paper (n = 193 nest fragments). The hydrocarbon concentration had a huge and significant increase (> 600%) during the 80-days sampling season (Fig 1). Initially, hydrocarbon concentration was similar in free-living and parasitized nests, but differed later on, so that parasitized nests had higher amounts of hydrocarbons than free-living nests at the end of the season (GLMM: nest status*time *F*_*1*,*165*_ = 6.452, P ≤ 0.01) (Fig 1). There were no significant effects of the number of brood (GLMM: *F*_*1*,*165*_ = 0.786; P = 0.377), population (GLMM: *F*_*1*,*165*_ = 0.229; P = 0.633) and colony phase (*F*_*1*,*165*_ = 0.013; P = 0.908).

### Analyses of the chemical signature of the nests

Interestingly, PCA showed that almost no linear alkanes (only two quantitatively minor ones) were involved in the 3 first components.

**Fig 2:**
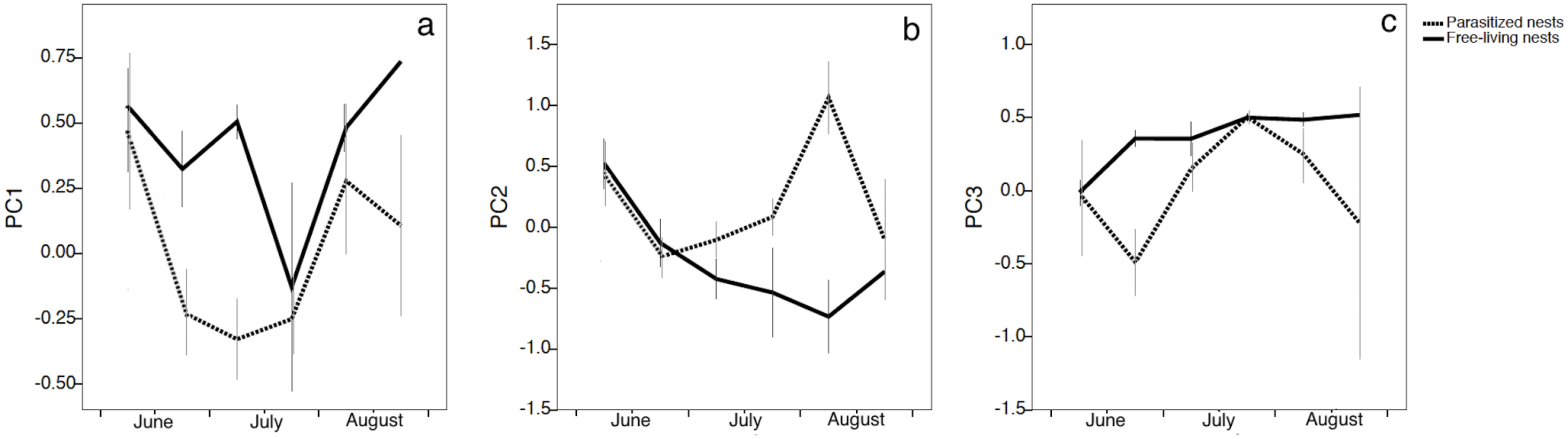
The variation in PC1 (a), PC2 (b) and PC3 (c) throughout the season in free-living nests controlled by the *P*. *biglumis* foundresses and in in parasitized nests controlled by the social parasite *P*. *atrimandibularis*.

PC1 explained 28% of variance and was strongly (positively) correlated with longer-chain hydrocarbons, that overall represented the 67% of the relative proportions of the longer-chain hydrocarbons in the signature; the only relatively lighter hydrocarbon loading on PC1 was 7-methylnonacosane. PC1 varied with time in different ways in free-living and parasitized nests, as shown by the significant interaction between nest status and time (GLMM: nest status*time *F*_*1*,*175*_ = 6.496; P = 0.012) (Fig 2a). PC1 values were relatively high during the first half of the colony cycle in free-living nests, then sharply declined, and increased again in August. In parasitized nests, PC1 values decreased as social parasites invaded, and were lower than those of free-living nests throughout the season (Fig 2a). These results suggest that the chemical signature of free-living nests was characterized by a relatively larger proportion of longer-chain hydrocarbons and 7-methylnonacosane (Peak 28), compared to the parasitized ones. There was no significant effect of the population, the number of brood and the colony phase on PC1 (brood *F*_*1*,*175*_ = 0.624; P = 0.431; population *F*_*1*,*174*_ = 0.048; P = 0.827; colony phase *F*_*1*,*175*_ = 2.661; P = 0.105).

PC2 (which explained 16% of variance) was strongly (positively) correlated with hydrocarbons with intermediate chain-length (i.e., 27–32 carbon atoms). PC2 values decreased regularly in free-living nests across the season, whereas they increased (after an initial decrease) in parasitized nests. This variation with time and nest status was significant (GLMM nest status*time: *F*_*1*,*176*_ = 9.839; P = 0.002) (Fig 2b). There were no significant effects of the population, the number of brood and the colony phase (brood *F*_*1*,*176*_ = 0.613; P = 0.435; population *F*_*1*,*174*_ = 0.152; P = 0.697; colony phase *F*_*1*,*175*_ = 0.307; P = 0.580).

**Fig 3:**
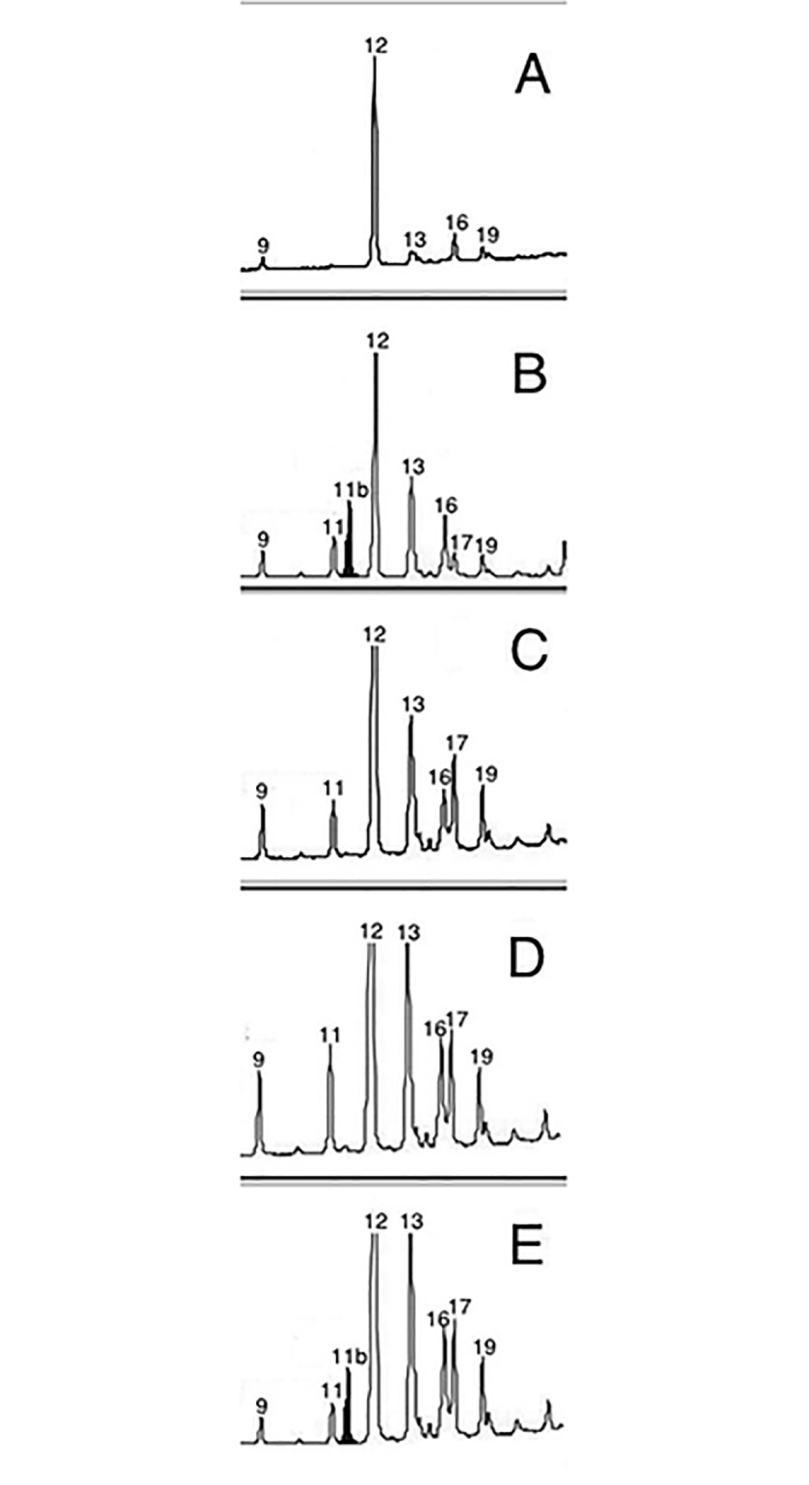
Representative gas chromatograms of a parasitized nest of *P*. *biglumis* at five different time points during the colony cycle (a: GC before parasite invasion; b: one week after parasite invasion; c: two weeks after parasite invasion d: three weeks after parasite invasion; e: six weeks after parasite invasion). Only hydrocarbons between peak 9 and peak 19 are shown. The picture shows the presence/absence of (Z)-9-heptacosene (the black peak), the most abundant alkene on parasitized nests. Peak 9: hexacosane; Peak 11: 2-methylhexacosane; Peak 11b: (Z)-9-heptacosene; Peak 12: n-heptacosane; Peak 13: 9-+11-+13-methylheptacosane; Peak 16: 11,15-dimethylheptacosane; Peak 17: 3-methylheptacosane; Peak 19: n-octacosane.

**Fig 4:**
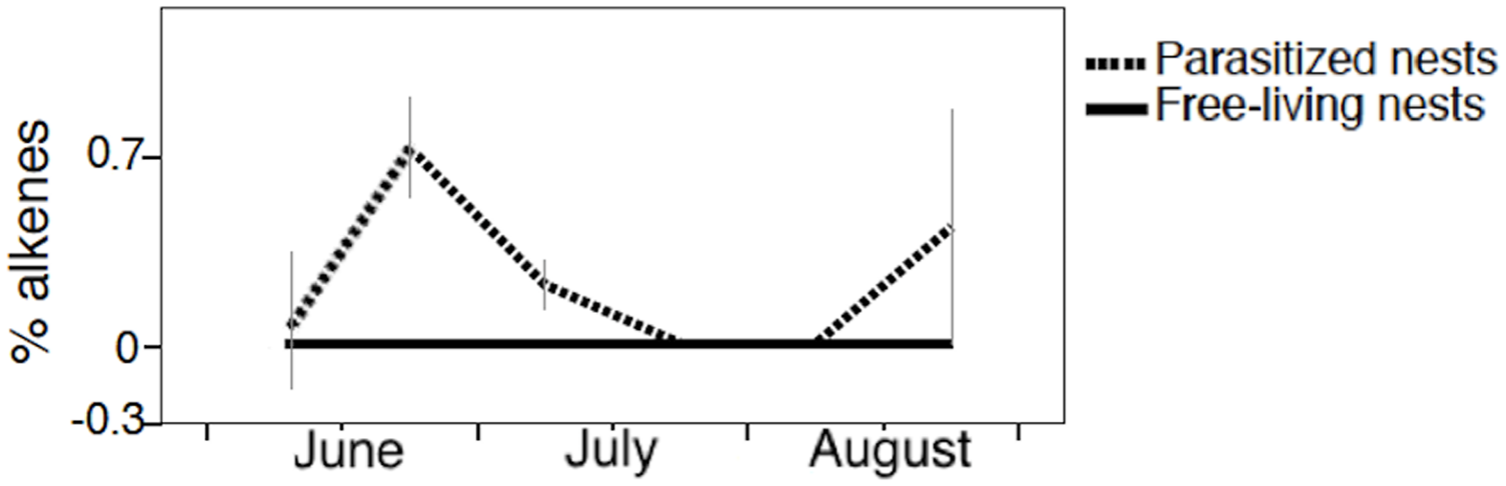
The variation in the proportion of alkenes on the nest paper throughout the season in free-living nests controlled by the *P*. *biglumis* foundresses and in parasitized nests controlled by the social parasite *P*. *atrimandibularis*.

The fate of PC3 (which explained 7% of variance) is especially interesting because the parasite-specific compounds loaded the most on this component. PC3 was strongly, negatively correlated with the alkenes (*Z)*-9-pentacosene (Peak 3a), (*Z)*-9-heptacosene (Peak 11b), the main alkene, and (*Z)*-9-nonacosene (Peak 26a), and positively correlated with 2-methylhexacosane (Peak 11) (Table 2). Fluctuations in PC3 values over time were mainly due to the changes in the proportions of these compounds (Fig 2c). Indeed, PC3 values varied significantly over time (*F*_*1*,*177*_ = 18.385; P < 0.0001), as a function of nest status (*F*_*1*,*177*_ = 13.170; P < 0.0001), and of the amount of brood in the nest (*F*_*1*,*177*_ = 4.511; P = 0.035). There was no significant effect of other factors (population *F*_*1*,*176*_ = 0.201; P = 0.655; colony phase *F*_*1*,*175*_ = 0.021; P = 0.886). PC3 values increased slightly during the summer in free-living nests. In parasitized nests, instead, PC3 values rapidly decreased when parasites invaded the nests, as we expected since the parasite-specific compounds were negatively correlated with this PC. Therefore, parasite-specific compounds contaminated nest paper. PC3 values in parasitized nests rapidly increased the next four weeks, indicating–because of the negative correlation–that parasite-specific compounds decreased in these nests towards mid-July (i.e., at the time when parasites are expected to mimic the chemical signatures of their hosts) (Fig 2c). When the parasite brood started to emerge, PC3 values changed again, suggesting that alkene proportions increased once more at the time the parasite offspring emerged. Indeed, alkenes were detectable in the gas chromatograms of parasitized nests at parasite invasion and in August (as shown for (*Z)*-9-heptacosene in Fig 3), when their relative proportions, although very small, were different from zero (Fig 4).

**Table 2:** The loading matrix (rotated component matrix) for the PC1, PC2 and PC3 (only peaks loading > 0.7 are shown). Peaks are sorted by loading size.

PEAK	HYDROCARBONS	PC1	PC2	PC3
61	2-Methyltetratriacontane	0.907		
63	9,23-+11,15+11,17-Dimethylpentatriacontane	0.884		
53	9-+11-+13-+15-+17-Methyltritriacontane	0.882		
42	7-Methylhentriacontane	0.851		
41	9-+11-+13-+15-Methylhentriacontane	0.851		
54	13,21-+11,15-Dimethytritriacontane	0.829		
55	9,19-+9,21-+9,23-Dimethyltritriacontane	0.829		
44	9,17-+9,19-+9,21-Dimethylhentriacontane	0.808		
43	11,15-Dimethylhentriacontane	0.808		
49	11,15-+11,19-+11,21-Dimethyldocotriacontane	0.797		
28	7-Methylnonacosane	0.740		
62	11-+13-+15-+17-Methylpentatriacontane	0.728		
32	5,11-+5,13-Dimethylnonacosane		0.926	
33	5,9-Dimethylnonacosane		0.926	
46	n-Docotriacontane		0.918	
47	3,9-+3,11-+3,13-Dimethylhentriacontane		0.918	
35	3,9-+3,11-+3,13-Dimethylnonacosane		0.896	
15	5-Methylheptacosane		0.840	
17	3-Methylheptacosane		0.835	
31	3-Methylnonacosane		0.826	
18	5,11-+5,13-Dimethylheptacosane		0.798	
45	5,11-+5,13-Dimethylhentriacontane		0.714	
58	n-Tetratriacontane		0.713	
59	3,9-+3,11-+3,13-+3,15-Dimethyltritriacontane		0.713	
29	5-Methylnonacosane		0.710	
3a	9-Pentacosene			-0.896
11b	9-Heptacosene			-0.890
26a	9-Nonacosene			-0.884
11	2-Methylhexacosane			0.833

### Analyses of the relative proportions of longer-chain and branched hydrocarbons

**Fig 5:**
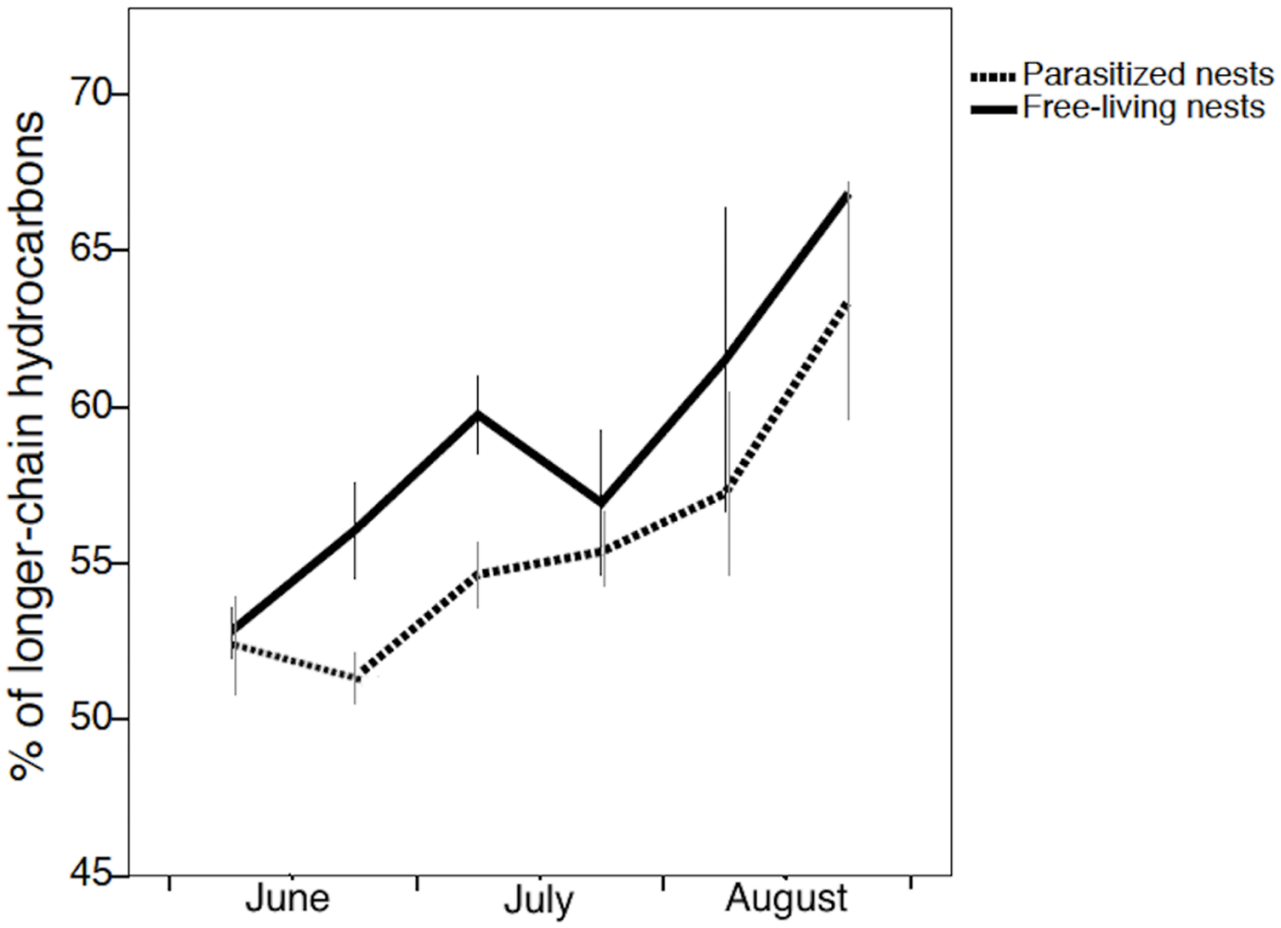
The variation in the proportion of longer-chain hydrocarbons on the nest paper throughout the season in free-living nests controlled by the *P*. *biglumis* foundresses and in parasitized nests controlled by the social parasite *P*. *atrimandibularis*.

The proportion of longer-chain hydrocarbons on the nest surface changed with time and nest status (GLMM: time *F*_*1*,*177*_ = 86.319; P < 0.0001; nest status *F*_*1*,*177*_ = 15.573; P < 0.0001). It increased during the season, but was consistently higher in free-living than in parasitized nests (Fig 5). The number of brood had a marginally significant effect (GLMM: brood *F*_1,177_ = 5.237; P ≤ 0.05), while population and colony phase (i.e., the presence or absence of adult offspring) had no significant effect (population *F*_1,175_ = 0.093; P = 0.761; colony phase *F*_1,176_ = 0.157; P = 0.692).

**Fig 6:**
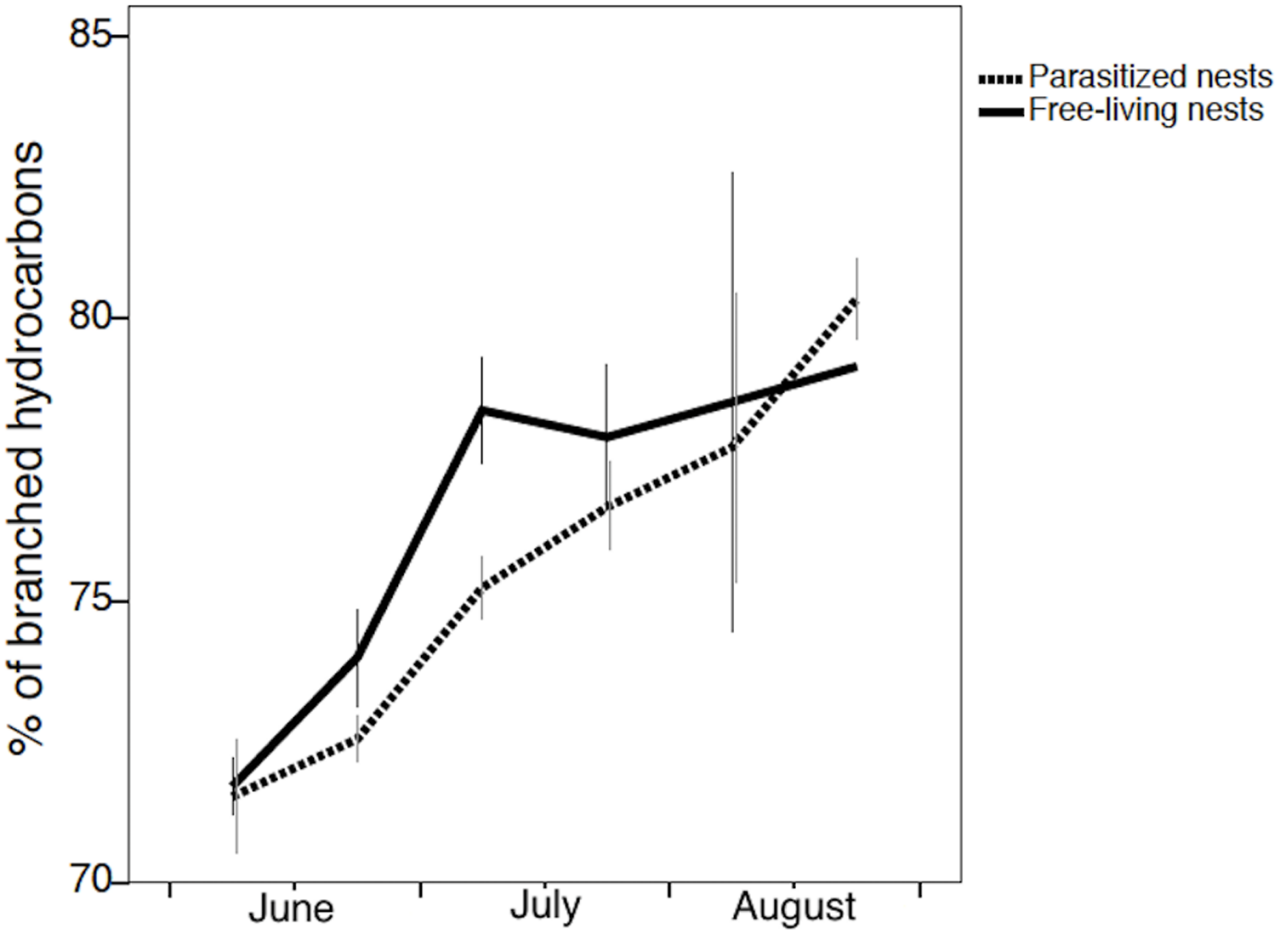
The variation in the proportion of branched hydrocarbons on the nest paper throughout the season in free-living nests controlled by the *P*. *biglumis* host foundresses and in parasitized nests controlled by the social parasite *P*. *atrimandibularis*.

The proportion of branched hydrocarbons increased over time as well (GLMM: time *F*_*1*,*188*_ = 67.572; P < 0.0001), and did not vary significantly between free-living and parasitized nests (nest status *F*_*1*,*188*_ = 2.129; P = 0.146) (Fig 6). Moreover, there were no significant effects of the population, the number of brood and the colony phase (brood *F*_*1*,*175*_ = 0.927; P = 0.337; population *F*_*1*,*188*_ = 1.629; P = 0.203; colony phase *F*_*1*,*188*_ = 1.255; P = 0.264).

## Discussion

Our results show that nests of the paper wasp *P*. *biglumis* were covered by a rich layer of hydrocarbons, which matched the composition and the relative proportions of the blends of cuticular hydrocarbons reported for the wasps of the same species [39, 40, 41]; (Elia unpubl. data). By tracking nest signature throughout colony cycle in two different populations, we found that the concentration of hydrocarbons, as well as the proportions of longer-chain and branched hydrocarbons, increased from the moment colonies were founded at the beginning of June to their decline at the end of August. These seasonal changes were modified when *P*. *biglumis* nests were invaded by obligate social parasites.

We analysed the chemical blend on the nest paper by repeated sampling. By doing this, we were able to follow for the first time how nest signature varied during the season in field colonies, where neither the adult wasp population, nor the brood were altered during the sampling procedure.

*P*. *biglumis* colony signature changed over time: on average, the amount of hydrocarbons on the nests increased seven times during about 80 days, which indirectly reinforces the hypothesis that the nest has a central role in the mechanisms of nestmate recognition in social wasps and open the question whether nests are similarly rich in hydrocarbons in hover wasps, which prioritizes visual cues over colony odor cues in nestmate recognition [47, 48]. A strong increase in hydrocarbon concentration with colony cycle has also recently found in bumblebee wax, where quantitative and qualitative variations in wax characteristics may affect worker reproductive decisions [49]. In contrast, in some ant, like *Camponotus aethiops*, the role of nest odor is not so crucial, and the colony signature on the nest walls is quantitatively scarce [10]. However, colony odor changes in ants as well, as shown in the weaver ant *Oecophylla smaragdina* [50] or in the ant desert *Cataglyphis niger*, where 25% of the hydrocarbons in the colony signature changed in relative proportions over a period of 3 months [51]. Similar results were shown by Provost et al. [52] in the ant *Temnothorax* (formerly *Leptothorax*) and in termites [53]. However, these studies tested changes in the colony signature of laboratory colonies, and here we test for the first time such changes in colonies in the wild throughout the nesting cycle.

It is unclear at the moment how the large quantitative changes in *P*. *biglumis* nest odor occurred, and focused experiments are needed. Since *Polistes* foundresses seem to be the main source of nest signature [13, 19], they may promote these variations through both active marking (as they often do by rubbing their abdomen on the nest surface) and repeated passive contacts with their nests. Similarly, Lenoir et al. [54], in experiments with the ant *Lasius niger* and Bos et al. [10] with the ant *Camponotus aethiops*, have shown that the hydrocarbon blends extracted from the soil covering ant-nest tunnels and chambers are deposited passively by ants via repeated contacts with the substrate. One of the main physico-chemical properties of cuticular hydrocarbons of social insects is their low volatility [9, 55, 56] and their long-term stability; cuticular hydrocarbons were still present on 20-year-old hornet specimens kept in a museum [57], as well as on 70-year-old specimens of scolytid cone beetles [58]. This may explain why the hydrocarbon concentration increased markedly over time on the *P*. *biglumis* nests; nest-marking and passive contacts of wasps with the nest surface continued throughout the summer. If the hydrocarbons were continuously deposited and did not volatilize, they accumulated and their concentration inevitably increased on the nest surface over time. The hypothesis that foundresses were mainly responsible for the production and maintenance of the nest signature in free-living colonies is further supported by the fact that the variations in nest signature during summer were not significantly associated with the emergence of the offspring in the nest (which began approximately in mid-July). Offspring emergence marks the end of the solitary founding phase in *P*. *biglumis* colonies and the beginning of a true social life, but the presence of newly emerged individuals did not significantly contribute to the increase in the amount of nest surface hydrocarbons.

As for the quality of the nest hydrocarbons, the values of PC1 (which represents mainly longer-chain hydrocarbons), the proportion of longer-chain hydrocarbons, and the proportion of branched hydrocarbons increased over the season. The values of PC2 (which represents mainly hydrocarbons with a chain length between 27–32 carbon atoms) decreased in the same period, as these hydrocarbons were overwhelmed by the important increase in the relative proportions of longer-chain hydrocarbons. Possibly, these changes on the nest were associated with similar changes on the foundress cuticle and/or in glands associated with nest-marking (e.g., Dufour’s or sternal glands). There is evidence that in newly emerged wasps, the relative proportions of some longer-chain hydrocarbons increased soon after emergence [59]. Similarly, mature wasps had higher relative proportions of branched and longer-chain hydrocarbons than younger ones [60, 61].

The variations in the colony signature over time, as represented by the nest signature, suggest that wasps constantly adjust their template to confine the acceptance range for reliable nestmate discriminations [2, 62]. Social insects may update their template via habituation to the updated signature; indeed, individuals exposed to non-nestmate signatures reduce their aggression to non-nestmates [11, 62, 63, 64].

The invasion of colonies by social parasites had many other important consequences on the nest signature in *P*. *biglumis* colonies, and produced several qualitative and quantitative changes, which suggests that parasites actively contribute to host nest signature.

The hydrocarbon concentration fluctuated in parasitized nests for most of the season, but it was smaller than in free-living nests at the end of the season. This is reasonable, because, although *P*. *atrimandibularis* parasites stroke their abdomen on host nests (at least soon after invasion) [18], they are chemically insignificant, and have approximately 20% the concentration of cuticular hydrocarbons of their hosts at. Parasite females at the end of their life cycle are no more chemically insignificant [65] and, if they mark, they may contribute to enrich the host nests in hydrocarbons.

While free-living and parasitized nests had similar proportions of branched hydrocarbons throughout the season, the proportion of longer-chain hydrocarbons was lower in parasitized than in free-living nests. Focused experiments are needed to investigate the causes of this difference. However, parasitized foundresses were subdued and inhibited by parasites in their egg-laying activity [18, 66], which might imply a decrease in the proportions of longer-chain hydrocarbons on their cuticular signature, as it occurs to subordinate foundresses relative to dominants [67].

Finally, parasitized nests differed strikingly from free-living nests in the presence of parasite-specific alkenes, as shown by the variations in PC3 values. We identified three alkenes on the nest surface, (*Z*)-9-pentacosene, (*Z*)-9-heptacosene, and (*Z*)-9-nonacosene, which are typical of the signature that parasites exhibit prior to mimicking their hosts [42]. We show that alkenes appeared on host nests the week after host-nest invasion, disappeared seven days later (Fig 3), and appeared again at the end of the season (Fig 4). Indeed, parasites may have deposited alkenes on the host nests through stroking [18] at invasion, when their chemical signature have alkenes, and again at the end of the season, when both the old parasites and their daughters possess them on their cuticle [5, 42]. We do not know if spreading the alkenes on the nest surface may serve any function, and if there is a function, why it is limited to short time periods (soon after host nest invasion, and at colony decline). Parasites interfere with the process of nestmate recognition of their host, because hosts make more recognition errors in parasitized than in living colonies (i.e., hosts are more tolerant towards non-nestmates and less tolerant towards non-nestmates)[43, 68]. The presence of alkenes might disrupt the learning of a “correct” template or modify the recognition threshold [43] and facilitates the acceptance of the parasite and/or her progeny in host colonies. Alternatively, alkenes might blurr parasite recognition cues, as it has been proposed for the incipient leaf-cutting ant *Acromyrmex echinatior* [69]. Another paper wasp social parasite, *P*. *sulcifer*, marks also the host nests with parasite specific compounds [21]. Such marking activity may diminish the chemical distance between the nest signature and the parasite signature [21]. Similarly, *intra*specific parasites cover host nests with their own signature, possibly to avoid unmasking by host workers and regulate host worker egg production [19, 20, 70].

Other social parasites produce compounds that reduce host aggression. For example, the males of the social parasite *Bombus vestalis* bumblebees excrete cephalic secretions that repel host workers [71], and the slave-maker ant *Polyergus rufescens* sprays ester decyl butyrate to reduce host aggression during colony usurpation [72]. The alkenes of *P*. *atrimandibularis* social parasites may have a similar role: even if strongly chemically insignificant [65], parasites are attacked by hosts when they land on host colonies, but then very rapidly submit their hosts [18]. Alkenes might reduce host aggression in the early stages of parasite invasion, and again at the end of colony cycle, when parasite offspring emerge. However, focused studies are needed to clarify the function of alkenes on parasitized nests and test whether they are an adaptive trait. Preliminary observations suggest that foundresses exposed to alkenes diminish their alarm response (Elia, unpubl. data.).

Overall, these findings may indicate that *P*. *atrimandibularis* social parasites, have a very elaborate chemical strategy of integration, based on marking host nests with parasite-specific hydrocarbons (this paper), being chemically insignificant [65] and achieving chemical mimicry [42].

We still lack a full comprehension of the hydrocarbon-mediated mechanisms that regulate interactions in social insect colonies. We have not yet revealed all of the complexities of how hydrocarbons function to regulate worker reproduction and to discriminate between intruders and legitimate colony members. Our study brings new and strong evidence that the nest signature changes in the wild, which has important consequences on the mechanisms of the learning of recognition cues, and their updating, and on the way we study learning and memory in social insects in general, and in parasitic context also. Our results bring new and original information about the dynamics of colony odor in undisturbed colonies in the wild and on the chemical-mediation of the behavior of hosts and parasites, and pose new areas of exploration on these topics.

## Supporting information

S1 Fig The chemical signature of nests of *P*. *biglumis* parasitised by the social parasite *P*. *atrimandibularis* or unparasitized (mean values +- s.e.obtained by pooling data across the nesting season, n = 193 nest fragments. (DOCX)

S2 Fig The two sampling site: Montgènevre in red, Ferrere in purple. (DOCX)
