# Supplementary material for: Nest signature changes throughout colony cycle and after social parasite invasion in social wasps
Source: PLoS One. 2017 Dec 19;12(12):e0190018. doi: 10.1371/journal.pone.0190018 (PMC5736209; doi:10.1371/journal.pone.0190018)
Supplement: S2 Fig — (DOCX) [file pone.0190018.s002.docx]

Nest signature changes throughout colony cycle and after social parasite invasion in social wasps

Marta Elia^1,2*^, Giuliano Blancato^1^, Laura Picchi^1^, Christophe Lucas^2^, Anne-Geneviève Bagnères^2 ¶^ , Maria Cristina Lorenzi^1,3 ¶^

*^1^Department of Life Sciences and Systems Biology, University of Turin, via Accademia Albertina 13, 10123 Torino, Italy*

*^2^I.R.B.I. – UMR 726 1CNRS – Université de Tours, Faculté des Sciences, Parc Grandmont, 37200 Tours, France*

*^3^LEEC-Laboratoire d'Ethologie Expérimentale et Comparée, Université Paris 13, Sorbonne Paris Cité, 99 avenue J.-B. Clément, 93430 Villetaneuse, France*

* marta.elia@outlook.com

^¶^ These authors contributed equally to this work


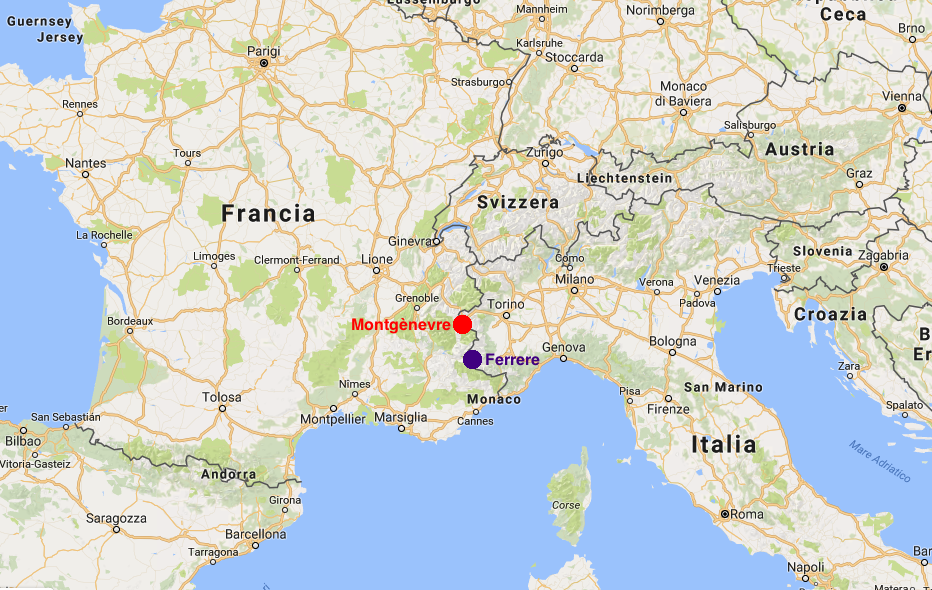


Figure S2 The two sampling site: Montgènevre in red, Ferrere in purple.
